# Examining the Convergent Validity of the Triarchic Psychopathy Measure Using a Sample of Incarcerated and on Probation Lithuanian Juveniles

**DOI:** 10.3390/bs9120156

**Published:** 2019-12-14

**Authors:** Laura Ustinavičiūtė, Alfredas Laurinavičius, Virginija Klimukienė, Ilona Laurinaitytė, Mykolas Baltrūnas

**Affiliations:** 1 Institute of Psychology, Vilnius University, 9/1 Universiteto St., 01513 Vilnius, Lithuania; alfredas.laurinavicius@fsf.vu.lt (A.L.); virginija.klimukiene@fsf.vu.lt (V.K.); ilona.laurinaityte@fsf.vu.lt (I.L.); mykolas@psichologas.lt (M.B.); 2 Institute of Psychology, Mykolas Romeris University, 20 Ateities St., 08303 Vilnius, Lithuania

**Keywords:** psychopathy, juveniles, TriPM, convergent validity

## Abstract

The Triarchic Psychopathy Measure (TriPM) is based on the triarchic psychopathy model proposed by Patrick, Fowles, and Krueger in 2009. This paper assesses the convergent validity of TriPM using a number of measures for a sample of adolescents who are either incarcerated or on probation. These included the Short-Term Assessment of Risk and Treatability: Adolescent Version (START: AV); the Subtypes of Antisocial Behavior Questionnaire (STAB); the Criminal Sentiments Scale-Modified (CSS-M); and the Measure of Criminal Social Identity (MCSI). The results showed significant differences between groups that are incarcerated and those on probation, with the incarcerated sample of juveniles exhibiting higher ratings in terms of Disinhibition and lower ratings for Boldness. The TriPM measures examined also show expected positive correlations with concurrent measures related to criminal behavior in both of the aforementioned samples of juveniles. A different pattern of correlations was observed between Boldness and STAB scales, with a large positive correlation found in the incarcerated sample, while no significant correlations were detected in the probation sample. The results support the usefulness of TriPM in assessing the psychopathy in samples of the juvenile offenders chosen for our research purposes.

## 1. Introduction

In the last 20 years, there has been a steady increase in published theoretical and empirical literature on psychopathy [1]. Psychopathy is associated with aggressive [2], violent criminal behavior [3,4,5] and with social and personal distress experienced by the victims of the psychopaths [1]. Psychology professionals working in the mental health and criminal justice systems regard this as one of the most important aspects of their work [1].

The study of juvenile psychopathy is less than two decades old [6,7], yet psychopathy contributes to the majority of general and serious crimes. It is only reasonable that there is increasing interest in the assessment of adolescents who exhibit psychopathic traits. Despite the fact that research in the area of juvenile psychopathy is rapidly expanding, it is still unclear what psychopathy in youth entails, and how it intersects with behaviors that are accepted as abnormal [6]. The early identification of youth who exhibit psychopathic traits could help us to arrest their descent into antisocial lives [8]. However, we must exercise caution when assessing psychopathy traits in youth, lest we assign a label that may be difficult to remove. The indiscriminate application of such concepts for an adolescent can have serious negative consequences [1,7], so we need to use appropriate, reliable, and valid measurements to assess youth psychopathy.

One of the most recent and frequently tested models is the Triarchic Psychopathy Model [6] which consists of three phenotypic constructs—Boldness, Meanness, and Disinhibition.

Boldness entails tendencies toward social dominance, thrill seeking/fearlessness, and low stress reactivity/emotional resiliency [6]. It is preferentially associated with narcissism, thrill/adventure seeking, and low inhibition system functioning [9] and with indices of adaptive function (stress immunity, social influence, lack of emotional instability) [10]. Meanness is best defined as a phenotypic manifestation of reduced empathic responding, callousness, exploitativeness, empowerment through cruelty, inability to form close relationships with others, and excitement seeking. Meanness reflects a tendency of aggressive resource seeking and having no regard to others [6]. Meanness is associated with low empathy, coldheartedness, egocentricity, and Machiavellianism [9]. Finally, disinhibition is mainly defined by impulse control difficulties and deficits, including nonplanfulness, failure to delay gratification, fun seeking behavior, irresponsibility, reactive angry emotionality, and social deviancy [6,9].

This model integrates a new approach to the psychopathy in comparison to historic writings [11]. In contrast to other psychopathy theoretical models it does not include criminal behavior as an essential component of psychopathy. The model was operationalized through the self-report Triarchic Psychopathy Measure (TriPM) questionnaire scales [12] that were designed specifically to index earlier introduced psychopathy traits [11]. The use of self-report measures of psychopathy is still debated [13], but the convergent and discriminant construct validity of the main scales of TriPM in relation to psychopathy relevant personality criteria is supported by the results of various studies in adult men and women samples, including community [9,11,13,14,15] and forensic and/ or correctional settings [9,11,13]. 

Research findings support the view that the scores of the TriPM Meanness and Disinhibition subscales correlate with aggression. Van Dongen and others (2017) examined TriPM in forensic and community adult samples and found that Meanness was more strongly associated with proactive aggression, whereas the Disinhibition scale was more strongly related to reactive aggression. No significant associations were found between Boldness and any type of aggression in their study [13]. The research of Fanti, Kyranides, Drislane, Colins, and Andershed (2016) within a sample of Greek-speaking non-clinical young adult females also confirm the links between callous unemotional characteristics of psychopathy and instrumental aggression. Boldness was associated with low hostility and verbal aggression, whilst Disinhibition was related to impulsive, irresponsible, and hostile tendencies; a relationship between physical aggression and Meanness was found [16]. The aforementioned confirms the premise that fearless-dominant tendencies of psychopathy are largely unrelated to aggression.

Weidacker, O’Farrel, Gray, Johnson, and Snowden (2017) examined the relationships between TriPM psychopathic traits and impulsivity across data obtained from prison and community participants. Disinhibition was related to high levels of negative/positive urgency and poor planning, while Boldness was related to high sensation seeking. A comparison of the samples showed small differences. Disinhibition in the incarcerated offender sample was greater than in the community sample, and sensation seeking was higher in the non-offender sample. The findings support the dimensional model of psychopathy, and some aspects of psychopathy in relation to reduced impulsivity were approved. Meanness was linked to most forms of impulsivity, and to low negative urgency and strong perseverance. According to Weidacker et al. (2017), this might explain the criminal behavioral peculiarities of some psychopathic offenders related to planning and persistence, since callousness might be related to instrumental violence, and this requires a high level of planning and persistence. However, we still do not have sufficient knowledge regarding the mechanisms underlying associations between psychopathy and crime to construct a viable explanation.

Pro-criminal attitudes are one of the greatest impact risk factors for criminal re-offending [17]. Prospero-Luis, Moreira, Paiva, Teixeira, Costa, and Almeida (2017) conducted their research in 91 adult male recidivist inmates convicted for theft and presented the evidence for associations between psychopathic traits and criminal attitudes for this sample. TriPM psychopathy fearlessness-related traits were associated with reduced expectancy of negative outcomes, and for Meanness in particular the increased expectancy of positive outcomes as a consequence of committing a crime. These abnormal appraisals of the probability of either being rewarded or punished results in criminal acts that lead to reoffending by psychopaths [18].

Nobody questions the link between serious offending and psychopathy, although, until recently, most scientific research focused on less severe deviant behaviors. This is beginning to change with an increasing body of research now being conducted among the general population on minor deviant behaviors (drug use, alcohol use, theft, vandalism, school misconduct, assault, and general deviance), and that are evaluated in relation to the TriPM constructs. A study by Coffey, Cox, and Kopkin (2018) [19] revealed the importance of TriPM to less severe deviant behavior. For example, all forms of normative deviance were positively predicted by Disinhibition, while Boldness positively predicted drug and alcohol use as well as general deviance. In addition, Boldness and Disinhibition positively predicted overall lifetime engagement in deviant behavior, and Meanness negatively predicted school misconduct.

The links between TriPM measured psychopathy dimensions and criminal behavior related correlates in adult samples confirm that TriPM is a reliable and valid psychopathy assessment tool. Notwithstanding, only a small number of studies have been conducted on the psychometric characteristics of TriPM using adolescent samples. Somma, Borroni, Drislane, and Fossati (2016) assessed the psychometric properties and construct validity of the Italian translation of the TriPM in three independent, Italian high school adolescents’ samples. Their results support the view that TRiPM is a reliable measure. TriPM also showed strong convergence with respect to the Psychopathic Traits Inventory [20] and high correlations to pro-social behavior, moral decision-making, and affective responding. Adolescents at high psychopathy risk (i.e., > 97 percentile on the TriPM) reported experiencing lower fear in response to emotion-eliciting movie clips in comparison to the low-psychopathy group. Similarly, hedonistic moral tendencies differentiated these two groups, so that higher moral tendencies in the low psychopathy group and negative associations between TriPM scores and Reflective Function Questionnaire were observed [21].

A recent study by Sadeh, Bounoua, and Javdani (2019) showed in a sample of 137 adolescents detained in juvenile detention facilities that TriPM psychopathic traits were significantly positively associated with symptoms of alcohol/substance use and anger/irritability, but only among youth who reported average-/late-pubertal development [22]. These findings implicate psychopathic personality traits as individual difference variables that may influence the onset of delinquent behavior. 

An examination of the Lithuanian translation of TriPM in an adult offender sample confirmed its construct validity in relationship to various risk assessment measures. The links between TriPM Boldness and PCL: SV Interpersonal facet, TriPM Meanness and the PCL: SV Affective facet, and TriPM Disinhibition and PCL: SV Lifestyle facet were found [23]. However, no research of the Lithuanian translation of TriPM was done to measure its validity in an adolescent group. 

The current study aimed to examine the convergent validity of the Lithuanian translation of TriPM by exploring links with psychopathy-relevant constructs for incarcerated and on-probation juvenile samples, as well as conducting a comparative analysis of TriPM measures in these two samples. On the basis of the literature review, the following hypotheses were formulated: (1) Meanness and Disinhibition should positively correlate with measures of aggression (STAB), pro-criminal attitudes (CSS-R, MSCI), and indicators of poor adaptation (START:AV Vulnerabilities) and negatively with variables indicating positive adaptation to the social environment (START:AV Strengths). (2) On the other hand, we expect that Boldness, the adaptive domain of the TriPM model, should be positively correlated to more successful adaptation (START:AV Strengths) and negatively to indicators of poor adaptation (START:AV Vulnerabilities). (3) As repeated and serious offending is positively related to Meanness and Disinhibition we expect that the incarcerated sample will be characterized by higher scores on these scales.

## 2. Materials and Methods

### 2.1. Participants

The data were obtained from two separate samples comprising a total of 189 male juveniles, an incarcerated sample (INC) (n = 30) and a sample that consisted of adolescents under the supervision of municipal probation offices (PS) (n = 159). The mean age of INC was 17.35 (SD = 0.53, range = 15.50–18.07) and for the PS was 16.95 (SD = 0.83, range = 14.37–18.29). The vast majority of the samples were Lithuanians. Youth were in the ninth school grade on average (in INC - M = 9.00, SD = 1.20; in PS-M = 9.56, SD = 1.16). Twenty-nine percent of the PS sample and 86% of INC had repeated the same school grade. Index offences included homicide (INC = 6.9%), physical violence/assault (INC = 3.4%; PS = 15.1%), sexual offence (INC = 3.4%; PS = 5.0%), robbery (INC = 41.4%; PS = 26.4%), theft (INC = 41.4%; PS = 28.9%), public order violation (INC = 3.4%; PS = 18.2%), vehicle offences (PS = 0.6%), drug offences (PS = 4.4%), and contraband (PA = 1.3%).

### 2.2. Procedure

The Ethical Committee of the Institute of Psychology at Vilnius University granted ethical approval No. 15 for this study on the 26 March 2018. The research was conducted in cooperation with the Prison Department under the Ministry of Justice of the Republic of Lithuania. Probation officers (PO) invited juveniles to participate in the study. POs contacted caregivers and received their active consent. Next, the juveniles provided their consent and filled in questionnaires. POs filled in demographic data questionnaires and were interviewed in vivo according to START: AV interview guidelines. On the basis of the interviews, the research team members made START: AV ratings. 

### 2.3. Measures

The Triarchic Psychopathy Measure (TriPM) [12] is a 58-item self-reported inventory that yields an overall psychopathy score along with three subscales. The Disinhibition scale evaluates the general propensity toward externalizing problems and comprises 20 items; the Meanness scale evaluates the callous aggression subdomain of the externalizing spectrum; and the Boldness scale evaluates the adaptive component of psychopathy entailing traits of dominance, emotional stability, and adventurousness. The latter two scales comprise 19 items each. The participants were asked to rate their agreement to each statement on a four-point scale: true (0); somewhat true (1); somewhat false (2); false (3). Cronbach’s *α* of TriPM was from 0.69 to 0.85.

The instruments described below were chosen as concurrent measures for psychopathy estimates, as their relationship with psychopathy is established in numerous studies cited above.

The Short-Term Assessment of Risk and Treatability: Adolescent Version (START: AV) [24] is a structured professional judgment scheme guiding the assessment of multiple adverse outcomes in adolescents between 13 and 18 years of age. In START: AV, there are 25 dynamic factors, each coded as Strengths (protective factors) and Vulnerabilities (risk factors) evidenced during the past three months on the three-point scale (0 = low, 1 = moderate, 2 = high). Cronbach’s *α* for Strengths was from 0.90 and for Vulnerabilities was 0.89.

The Subtypes of Antisocial Behavior Questionnaire (STAB) [25] measures a self-reported history of delinquent behavior and contains the following three scales: Physical Aggression (PA), Social Aggression (SA), and Rule Breaking (RB), consisting of 10, 11, and 11 items, respectively. Participants completed the STAB reporting if the indicated behavior has occurred at any time in their life. Cronbach’s *α* of STAB ranged from 0.83 to 0.93.

The Criminal Sentiments Scale—Modified (CSS-M) [26] is designed to measure three general categories of criminal attitudes. It consists of 41 items. The first 25 items comprise the subscale of Attitudes Towards the Law, Court, and Police (LCP); the next 10 items comprise the subscale of Tolerance for Law Violations (TLV), and the next 6 items comprise the subscale of Identification with Criminal Others (ICO). Cronbach’s *α* of rest into analysis included CSS-M scales ranged from 0.52 to 0.89.

The Measure of Criminal Social Identity (MCSI) [27] is an eight-item self-report measure. Responses are recorded on a five-point Likert scale (1 = “strongly disagree” to 5 = “strongly agree”), with scores rating from eight to 40. The scale included items measuring the level of personal bonding with other criminals, the psychological salience of a criminal’s group identity and a criminal’s felt attitude toward other in-group criminals. High scores of the MCSI indicate that criminal identity is crucial for an individual’s self-concept. Individuals with increased MCSI scores are likely to approve of and behave in a manner consistent with the group norms, even in the absence of group members. Cronbach’s *α* of MCSI was 0.57.

The demographic questionnaire was developed to gather the socio-demographic information about the research participants and their previous delinquent behavior. The questions related to the age, place of residence, school grade, family structure, and age of the first contact with police, etc. Probation officers on the basis of the case records filled in this questionnaire.

### 2.4. Statistical Analysis Methods

The SPSS 24.0 software package was used for statistical calculations. The Pearson’s *r* correlation coefficient was used for estimation of relationships between variables. When interpreting the strength of correlations, threshold values recommended by Cohen (1992) were as follows: *r* ≥ 0.10—low effect, *r* ≥ 0.30—medium effect, *r* ≥ 0.50—large effect.

The Student *t* test was used for intergroup comparisons. Effect sizes were calculated for normally distributed data, taking into account the standard deviations of the samples being compared. The interpretation of effect sizes was based on values recommended by Cohen (1992): *d* ≥ 0.20—small effect size, *d* ≥ 0.50—medium effect size, *d* ≥ 0.80—large effect size. 

## 3. Results

In order to test the convergent validity of the TriPM psychopathy measure, we investigated the associations between TriPM and psychopathy related measurements. Table 1 presents data for juveniles’ START Strengths, Vulnerabilities, Aggression, Criminal Sentiments, Criminal Social Identity, and their links to TriPM. Statistically significant relationships between the juveniles’ START: AV Strengths, Vulnerabilities, and TriPM psychopathy traits were identified only in the sample for on-probation supervision. Negative associations were found between Vulnerabilities and Boldness (*r* = −0.19, *p* < 0.05), and between juvenile’s Strengths and Disinhibition (*r* = −0.22, *p* < 0.05), while positive links were estimated between juveniles’ Strengths and Boldness (*r* = 0.19, *p* < 0.05), and Vulnerabilities and Disinhibition (*r* = 0.26, *p* < 0.01). However, the estimated correlations were of low effect (0.10 < *r* < 0.30).

Table 1 demonstrates the TriPM and the STAB scales’ correlation analysis is present in both samples of juveniles. We identified a large number of statistically significant positive correlations of medium and large effect sizes (*r* ≥ 0.30—medium effect, *r* ≥ 0.50—large effect). 

We found positive correlations of medium and large effect between the TriPM Boldness and STAB scales in the incarcerated sample—STAB total (*r* = 0.44, *p* < 0.05), Physical Aggression (*r* = 0.56, *p* < 0.01), and Rule Breaking Behavior (*r* = 0.37, *p* < 0.05). In the juvenile samples for those on probation we detected no significant relationships between Boldness and STAB scales. We test the differences of correlations in two samples. In the incarcerated sample there was a significantly higher correlation between Boldness and the STAB total (*z* = 2.55, *p* = 0.01), Physical Aggression (*z* = 3.08, *p* < 0.001), and Rule Breaking (*z* = 2.1, *p* = 0.036). 

As evident in Table 1, in the incarcerated sample TriPM Meanness and Disinhibition scales were associated with STAB total (*r* = 0.47, *p* < 0.01; *r* = 0.49, *p* < 0.01), Physical Aggression (*r* = 0.42, *p* < 0.05; *r* = 0.46, *p* < 0.05) and Rule Breaking Behavior (*r* = 0.47, *p* < 0.01; *r* = 0.50, *p* < 0.01). The correlations were of medium effect (0.30 ≤ *r* ≤ 0.50). In the probation sample we observed the expected correlations between TriPM Meanness and Disinhibition scales and STAB total (*r* = 0.51, *p* < 0.01; *r* = 0.64, *p* < 0.01), Physical (*r* = 0.57, *p* < 0.01; *r* = 0.53, *p* < 0.01), Social Aggression (*r* = 0.35, *p* < 0.01; *r* = 0.53, *p* < 0.01), and Rule Breaking Behavior (*r* = 0.34, *p* < 0.01; *r* = 0.62, *p* < 0.01) scales. Most of the correlations, except Meanness and Social Aggression and Rule Breaking Behavior associations, were of large effect (*r* ≥ 0.50).

The results shown in Table 1 also demonstrate the relationship of TriPM psychopathy scales to attitudinal measures (CSS-M). The observed relationships between TriPM scales and both attitudinal scales indicate that psychopathy is related to antisocial attitudes in criminal samples. The most pronounced relationship was observed between the Meanness and CSS-M total scales scores (*r* = 0.72, *p* < 0.01) in juveniles in the probation sample (*r* ≥ 0.50).

A comparative analysis of the TriPM psychopathy scales in incarcerated and probation samples demonstrate that both samples differ from each other on Boldness (*p* = 0.013) and Disinhibition (*p* < 0.001) (see Table 2). Boldness scored higher in the probation sample (medium effect size *d* = 0.46), whereas Disinhibition scored higher in the incarcerated sample (large effect size *d* = 1.09). No significant difference between the groups on Meanness scale was observed. In order to decrease the potential for family wise error, a Bonferroni correction was used. The a priori alpha level for each juvenile group was adjusted by dividing 0.05 by the number of TriPM scales (i.e., 0.05/3 = 0.017). The results from the *t* tests were considered further only if *p* < 0.017, which increased confidence that observed mean differences were unlikely to be due to chance.

For the most part, the results based on correlational and comparative analysis have confirmed our predictions based on literature review. 

## 4. Discussion and Conclusions

The purpose of our research was to test the convergent validity of TriPM in incarcerated and probation juvenile samples. The findings for the TriPM scales were generally consistent with the results of previous research. 

Disinhibition and Meanness were positively associated with aggression, rule breaking behavior, and pro-criminal attitudes in incarcerated and probation juvenile samples. Current research outcomes support the results of TriPM in forensic and community adult samples [15,16]. The findings correspond to explanations put forward by Prospero-Luis and colleagues (2017) that psychopathy is related to the positive evaluation of the consequences of committing a crime. Conversely, a different pattern of correlation was observed between boldness and physical aggression, rule breaking behavior, and pro-criminal attitudes. A strong association with physical aggression was found in the incarcerated sample, while no links were detected in the probation sample. In addition, the correlations in both samples differ quite significantly, which indicates the moderating effect of the group (incarceration vs. probation), where belonging to a more antisocial group strengthens the relationships between boldness and aggressive behavior. Physical aggression in the incarcerated sample can be used as an instrument to gain approval, achieve a higher status, and to adapt to a custodial environment where aggressiveness is valued. However, results for the probation sample support the premise that the emotionally stable, fearless-dominant tendency of psychopathy is largely unrelated to aggression [16]. The positive link of boldness to the strengths and negative link to the vulnerabilities measured by START: AV in the probation sample was observed. The results of our study confirm that boldness is an adaptive domain of the TriPM model in a less criminalized sample, or it can point to more antisocial individuals in samples characterized by strong antisociality. On the other hand, Disinhibition was positively linked to indicators of poor adaptation, and negatively to the strengths of juveniles under probation supervision. It supports the general propensity of this psychopathy trait towards externalizing problems in the probation juvenile sample.

Our study results offer partial support to the third research hypothesis. Significant differences between incarcerated and probation groups were found, such that samples from incarcerated juveniles showed higher ratings on the Disinhibition scale and lower on the Boldness scale. It was expected that the incarcerated sample would score more highly on Meanness and Disinhibition. Greater Disinhibition in the sample of incarcerated offenders in comparison to the community sample is supported by previous research [28] and shows that juveniles under probation supervision in comparison to incarcerated adolescents are more self-confident, more emotionally stable, and have a greater chance of remaining in the community. Moreover, the juvenile who has significantly worse control over their impulses has a greater probability of being incarcerated.

Recent study tested the relevance of the Triarchic psychopathy model in a population of juvenile offenders. The research results of TriPM have shown most of the expected relationships with concurrent measures related to criminal behavior. The study results appear to demonstrate that TriPM may be used for further research purposes for the assessment of juvenile psychopathy.

## Figures and Tables

**Table 1:** Correlations between Triarchic Psychopathy Measure (TriPM) and convergent measures.

Concurrent Measures	TriPM					
	Boldness		Meanness		Disinhibition	
	INC	PS	INC	PS	INC	PS
START: AV						
Strengths Total	0.16	0.19 *	−0.13	−0.17	−0.13	−0.22 *
Vulnerabilities Total	−0.16	−0.19 *	0.22	0.09	0.27	0.26 **
STAB Total	0.44 *	−0.06	0.47 **	0.51 **	0.49 **	0.64 **
Physical Aggression	0.56 **	−0.01	0.42 *	0.57 **	0.46 *	0.53 **
Social Aggression	0.25	−0.11	0.35	0.35 **	0.35	0.53 **
Rule Breaking	0.37 *	−0.05	0.47 **	0.34 **	0.50 **	0.62 **
CSS-M Total	0.30	0.19 *	0.72 **	0.54 **	0.55 **	0.28 **
LCP	0.19	0.22 **	0.65 **	0.49 **	0.48 **	0.22 **
TLV	0.39 *	0.13	0.70 **	0.43 **	0.52 **	0.22 **
ICO	0.41 *	−0.01	0.64 **	0.52 **	0.56 **	0.41 **
MCSI Total	0.23	0.05	0.46 *	0.41 **	0.32	0.34 **

*Note.* INC—incarcerated juvenile sample (*n* = 30), PS—under probation supervision sample (*n* = 159); START: AV = Short-Term Assessment of Risk and Treatability: Adolescent Version; STAB = Subtypes of Antisocial Behavior Questionnaire; CSS-M = Criminal Sentiments Scale-Modified; LCP = Law-Court-Police; TLV = Tolerance for Law Violation; ICO = Identification with Criminal Others; MCSI = Measure of Criminal Social Identity. Statistically significant correlations are bolded. * *p* < 0.05 (2-tailed), ** *p* < 0.01 (2-tailed).

**Table 2:** Comparison of TriPM measures between incarcerated and on probation juvenile samples.

	Incarcerated sample (n = 30)	Probation sample (n = 157)			
	*M (SD)*	*M (SD)*	*t*	*p*	*d*
**Boldness**	**26.30 (8.59)**	**29.91 (6.97)**	**2.500**	**0.013**	**0.46**
Meanness	16.90 (8.66)	17.10 (9.27)	0.110	0.912	0.02
**Disinhibition**	**31.87 (9.73)**	**21.18 (9.81)**	**−5.477**	**< 0.001**	**1.09**

*Note*. Statistically significant mean differences (*p* < 0.017) are bolded.
